# The Clinical and Economic Burden of Chronic Kidney Disease in Poland: Inside Patient-Level Microsimulation Modelling of CKD

**DOI:** 10.3390/jcm14010054

**Published:** 2024-12-26

**Authors:** Anna Masajtis-Zagajewska, Renata Kurek, Katarzyna Modrzyńska, Timothy Coker, Michał Nowicki

**Affiliations:** 1 Department of Nephrology, Hypertension, Transplantation and Internal Medicine, Central University Hospital, Medical University of Lodz, 90-419 Lodz, Poland; anna.masajtis-zagajewska@umed.lodz.pl; 2 Medical Affairs, Cardiovascular Renal and Metabolism (CVRM), BioPharmaceuticals, AstraZeneca Europe and Canada, 31-503 Krakow, Poland; renata.kurek@astrazeneca.com (R.K.); katarzyna.modrzynska@astrazeneca.com (K.M.); 3 HealthLumen Limited, London EC3N 2PJ, UK

**Keywords:** chronic kidney disease (CKD), economic burden, epidemiological impact

## Abstract

**Background/Objectives:** Chronic kidney disease (CKD) is associated with increased annual costs, with the highest costs attributable to renal replacement therapy (RRT). These costs will rise as prevalence increases. Therefore, forecasting the future prevalence and economic burden of CKD, particularly in underdiagnosed populations, may provide valuable insights to policymakers looking at strategies to implement interventions to delay CKD progression. **Methods**: As part of the Inside CKD study, this work used epidemiological data to generate a virtual population representative of Poland that progressed through a microsimulation in 1-year increments between 2022 and 2027. This microsimulation was used to assess the clinical and economic burdens of CKD in Poland. **Results**: Between 2022 and 2027, the percentage of individuals with CKD is projected to increase from 10.7% to 11.3%. Only 30.1% of individuals with CKD will be diagnosed in 2027. During this time, the total healthcare cost of individuals with diagnosed CKD pre-RRT is predicted to decrease slightly from $73 million to $62 million. However, the total healthcare cost of individuals with diagnosed CKD is projected to increase by 23.1% when including RRT. **Conclusions**: This study shows that the clinical and economic burdens of individuals with CKD will worsen in the upcoming years. The implementation of policies to enhance the early detection of CKD and the initiation of treatments to slow disease progression should be implemented to reduce the number of individuals requiring RRT.

## 1. Introduction

Chronic kidney disease (CKD) is a progressive and irreversible condition affecting >10% of the world’s adult population [1,2,3]. CKD is associated with significant morbidity and mortality, ranking as the third-fastest-growing cause of death globally [2,4]. Epidemiological data on CKD in Poland is scarce. In the 2011 nationwide NATPOL study, analyzing the prevalence and control of heart disease risk factors in Poland, 5.8% of the participants had CKD, and the estimated prevalence of CKD in the Polish population aged 18–79 years was close to 2 million, although some studies of global data suggest that CKD may affect up to 4 million individuals in Poland [3,5,6].

Most recommendations for assessing CKD focus on high-risk groups, such as individuals with diabetes or hypertension [7]. According to the National Health Fund, CKD has been diagnosed in 210,000 Poles [8]. However, the National Health fund mostly reports cases involving patients under continuous nephrology care and/or eligible for renal replacement therapy (RRT), and therefore may be underestimating the number of cases of diagnosed CKD in Poland [6]. This is in sharp contrast with the projections of at least 4.8 million people with diagnosed CKD living in Poland [9]. One possible reason for the low rate of CKD diagnosis could be a lack of awareness among the public and doctors regarding the symptoms, causes, risk factors, and treatment of CKD. Individuals with early stages of CKD are often unaware of its presence due to its asymptomatic nature [10], leading to the delayed recognition and implementation of early interventions that could slow down disease progression, delay complications, extend life expectancy, and consequently reduce the economic burden on the healthcare system. Diagnosis in many individuals occurs only in the advanced stages of the disease when alarming clinical symptoms appear, and often the only proposed treatment method is RRT.

Screening tools for CKD are easy to implement and cost-effective. The Kidney Disease: Improving Global Outcomes (KDIGO) guidelines from 2012 recommend classifying CKD patients into six categories (G1, G2, G3a, G3b, G4, and G5) based on estimated glomerular filtration rate (eGFR) and three categories (A1–A3) based on the level of albuminuria [11,12,13]. The combined assessment of eGFR and albuminuria allows for a more precise estimation of CKD prevalence, progression risk, and complications and facilitates better decision-making regarding patient monitoring and treatment [11,14,15]. Numerous studies indicate that screening programmes can be cost-effective, making their implementation highly beneficial [16,17,18].

CKD is associated with significant healthcare costs, especially in relation to RRT in the final stage of CKD [19]. Awareness of the need to prevent CKD progression necessitates the development and implementation of increasingly improved guidelines on early detection strategies and continuous care for patients with CKD. Therefore, the early detection of kidney disease, the early implementation of conservative treatment to slow down disease progression, reduce complications, improve individuals’ quality of life, and lower costs associated with CKD and its complications are of the utmost importance. This is crucial considering the ageing population of Poland and the increasing prevalence of CKD-associated comorbidities, such as diabetes and hypertension.

Cardiovascular diseases are also highly prevalent in individuals with CKD, which represent another significant economic burden [9,20]. Additionally, CKD is associated with increased mortality, more frequent healthcare utilization, and a higher risk of hospitalization [21,22,23]. As a result, the annual direct all-cause healthcare costs for CKD are extremely high and increase with the rising prevalence of CKD [24]. Thus, the economic burden of caring for individuals with CKD is substantial for both providers and patients.

By forecasting the future prevalence and diagnosis rate of CKD, this study aims to provide insights into both the clinical and economic burdens of CKD in Poland in the long term.

## 2. Materials and Methods

### 2.1. Overview of Model and Clinical Burden

The Inside CKD study used demographic and epidemiological data from Poland to generate a virtual population representative of the Polish population that progressed through a microsimulation in 1-year increments; detailed methods have been published previously [25,26,27,28]. The individuals within the simulated populations were assigned baseline characteristics (sex, age, and body mass index [BMI]) according to national statistics. They were also given an eGFR and albuminuria status at baseline, based on data from national health and epidemiological surveys. These clinical characteristics differentiated individuals without CKD from the CKD cohort, with the latter stratified by CKD stages 1–5 according to KDIGO (Kidney Disease Improving Global Outcomes) guidelines [11]. Some individuals with CKD stage 5 could, according to the guidelines of the country under consideration, receive renal replacement therapy (RRT) and thus represented a subgroup within the CKD stage 5 population. Those with CKD could also be assigned relevant comorbidities and/or complications on the basis of the known prevalence of each condition in the country-specific CKD population [25,26,27,28].

Virtual individuals were cycled annually through the microsimulation and aged each year from baseline to the final year. The data inputs (described in the modules below) defined the likelihood of a change in health state for any given individual. The probability that CKD emerged in individuals who were disease-free at baseline was informed by background eGFR decline and albuminuria in the general population.

For individuals already diagnosed with CKD, the probability of progression was estimated using eGFR and urine albumin creatinine ratio (ACR) status, both of which were updated annually. As eGFR declined and/or ACR increased—both were modelled in accordance with epidemiological data. Individuals with comorbidities at baseline (and those who acquired comorbidities later in the model) had a rate of progression ascertained according to annual regression slopes [25,26,27,28].

This microsimulation was used to assess the clinical and economic burdens of CKD in Poland. The model was used to define the likelihood of a change in health state for each individual between 2022 and 2027 (Tables S1–S4). Where data were unavailable, data from Greece, Netherlands, Romania, the UK, or Australia were used as a proxy. These data were assumed to be the most similar to Poland, compared to other available sources, and all input data were validated by local clinical experts.

A virtual population of 20 million individuals representative of the overall Polish population (with and without CKD) was created for 2022. The results were then rescaled to ~37.7 million to match the population of Poland in 2022. The input data were used to assign baseline characteristics including age, sex, eGFR, urine albumin–creatinine ratio, CKD status (no CKD, or CKD stage 1–5), the risk of developing (or the presence of) cardiovascular complications, comorbidities (type 2 diabetes, hypertension), and the risk of all-cause death. As virtual individuals were cycled annually through the microsimulation, the risk of developing CKD (in those without CKD), CKD progression (in those with CKD), developing cardiovascular comorbidities, and all-cause death were adjusted according to an individual’s characteristics. For the risk of CKD progression, the annual declines in eGFR were estimated using data from the DISCOVER CKD global observational study, as described previously [28].

Sensitivity analyses of key parameters were conducted to evaluate the robustness of potential findings to changes in input data and assumptions, in particular, eGFR slopes and diagnosis rates.

### 2.2. Economic Burden

Healthcare costs associated with the management of CKD and associated complications were limited to individuals with diagnosed CKD stages 3–5. Costs for CKD stages 1–2 were not estimated to avoid potentially confounding costs associated with conditions other than CKD. Economic data from Poland including costs of CKD, RRT (haemodialysis, peritoneal dialysis, and kidney transplants), and associated complications were inputted into the Inside CKD model and validated by local clinical experts (Table S5). Cost estimates were adjusted to 2022 prices according to the gross domestic product deflator data from the International Monetary Fund (IMF). Where costs were originally reported in other currencies, they were converted to Polish zloty (PLN) using a purchasing power parity method based on the IMF deflator data [28]. All cost outputs were subsequently converted from PLN to international dollars using the same method.

Country-specific unit costs were corrected for inflation between the year the data were collected and the start year of the microsimulation. To address country-specific data gaps, unit costs were converted to the relevant local currencies using precise point positioning algorithms. The future costs and outcomes of healthcare interventions were discounted to present values, adjusting for differences in expenditure compared with outcomes. The model included direct costs associated with CKD stages (costs for CKD stages 1 and 2 were assumed to be zero) and RRT costs to calculate the economic burden [25,26,27,28].

### 2.3. Statement of Ethics

This study was conducted in accordance with ethical principles of the Declaration of Helsinki and Good Clinical Practice guidelines. The Inside CKD study did not involve human participants and therefore did not require informed consent or institutional/ethical review board approval.

## 3. Results

### 3.1. Baseline Characteristics

The baseline characteristics of the total Poland population used in the model to project the clinical and economic burdens of CKD are shown in Table 1. Males made up 48.4% of the population and 20.0% were aged ≥65 years. The prevalence of CKD was 10.7%, with 5.9% having CKD stage 3, 0.2% having CKD stage 4, and <0.1% having CKD stage 5. Of individuals with CKD, 17.1% had heart failure, 57.3% had hypertension, and 20.0% had type 2 diabetes.

### 3.2. Clinical Burden of CKD

**Figure 1:**
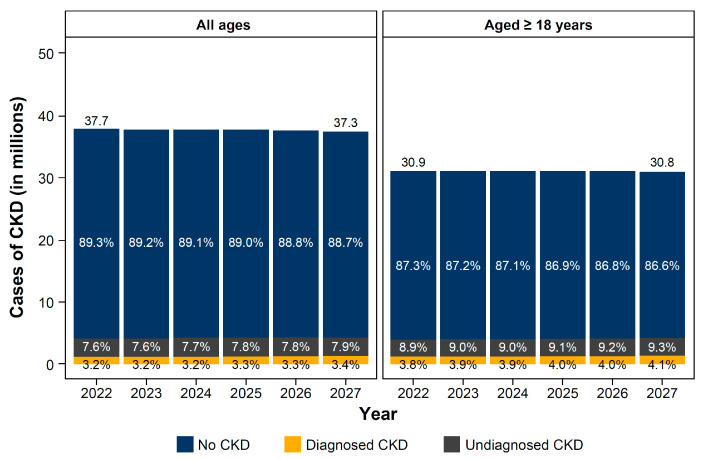
Percentage of the population with CKD in Poland, by age categories. CKD, chronic kidney disease.

**Figure 2:**
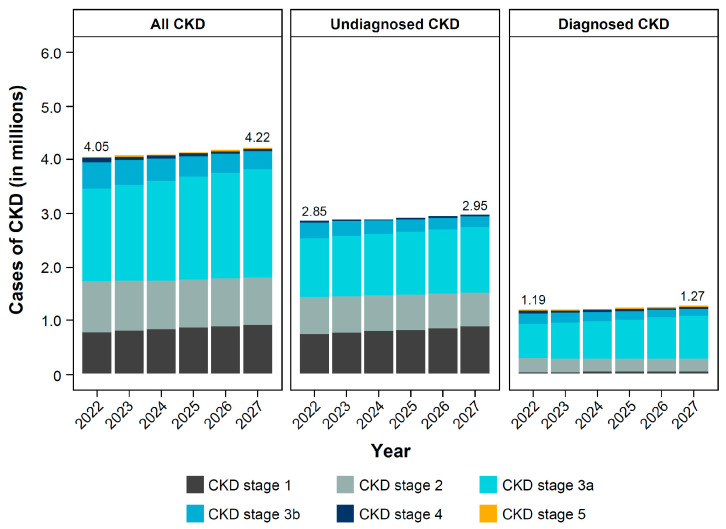
Number of cases of CKD in Poland, by stage. CKD stage 5 contains individuals on renal replacement therapy. CKD, chronic kidney disease. © CC BY 2024. Figure adapted from: Projecting the clinical burden of chronic kidney disease at the patient level (Inside CKD): a microsimulation modelling study. G. Chertow et al. (Appendix 1 [Poland]) [26]. This work is licensed under a Creative Commons Attribution License https://creativecommons.org/licenses/by/4.0/.

Between 2022 and 2027, the percentage of individuals with CKD is projected to increase from 10.7% (4.0 million) to 11.3% (4.2 million), despite a slight decrease (by 0.425 million) in the overall Polish population (Figure 1). This rise in cases of CKD was also observed in the population aged ≥18 years (12.7% in 2022 to 13.4% in 2027). The percentage of individuals receiving a CKD diagnosis in the overall population is expected to remain low, and based on the current clinical practice, only 30.1% (1.3 million) of individuals with CKD (all stages) will have received a diagnosis by 2027 (Figure 2). The majority of individuals with CKD (diagnosed and undiagnosed) are predicted to have CKD stage 3 (56.0% in 2027), while only a minority of individuals will have late-stage CKD (1.4% of stages 4–5 in 2027).

Individuals with CKD stages 1–2 tend to be undiagnosed, making up 51.4% of the undiagnosed CKD population in 2027 (Figure 2). In comparison, CKD stage 1–2 represent 22.0% of the diagnosed CKD population in 2027. Only 39.8% of individuals with CKD stage 3 will be diagnosed, making up 74.2% of all individuals diagnosed with CKD in 2027.

**Figure 3:**
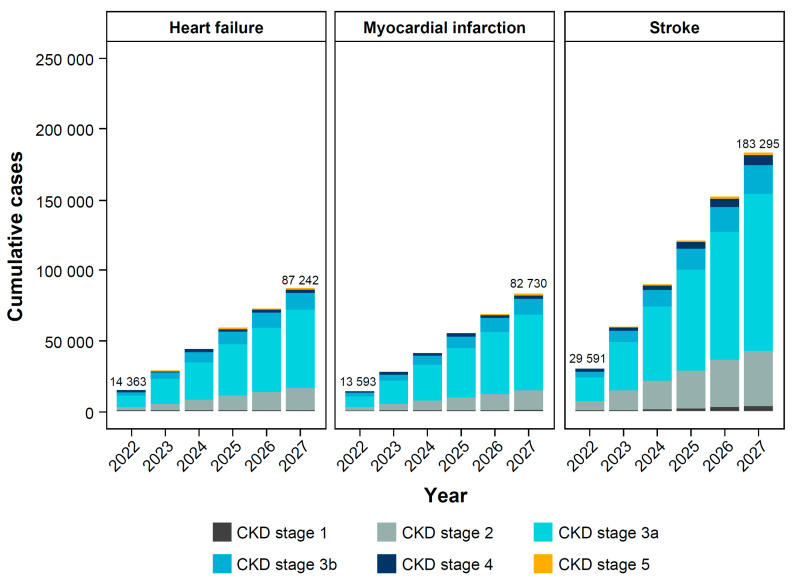
Projected complications in individuals with diagnosed CKD. CKD, chronic kidney disease.

**Figure 4:**
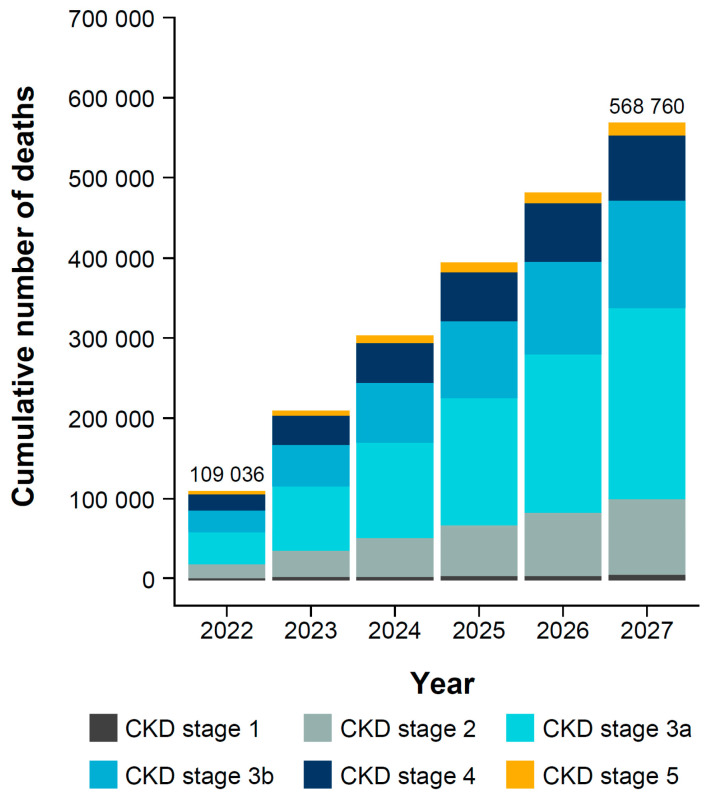
Projected cumulative all-cause deaths in individuals with diagnosed CKD, by stage and year. CKD, chronic kidney disease.

There are predicted to be 353,267 new cases of CKD complications (heart failure, myocardial infarction, or stroke) in the diagnosed CKD population by 2027 (Figure 3). The prevalence per 100,000 individuals with CKD is projected to reach 14,501 for heart failure, 17,762 for myocardial infarction, and 44,838 for stroke by 2027. In those with diagnosed CKD, 568,760 all-cause deaths are expected by 2027 (Figure 4, Table S6). The majority of these all-cause deaths will be in individuals with CKD stage 3 (67.6% in 2027).

### 3.3. Economic Burden of CKD

**Figure 5:**
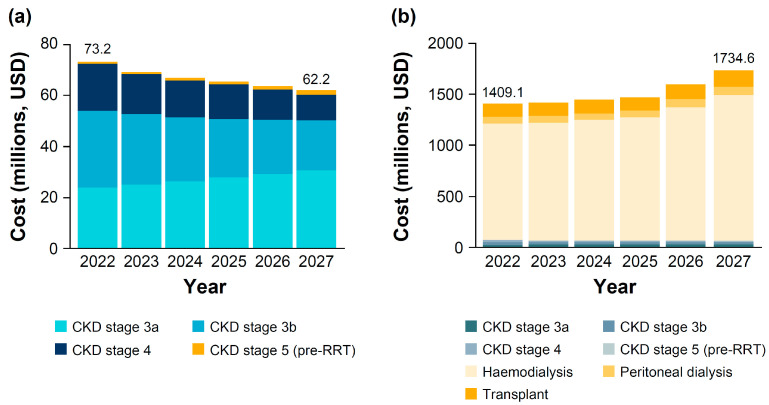
(**a**) Total healthcare costs associated with diagnosed CKD (pre-RRT); (**b**) healthcare costs associated with diagnosed CKD and RRT. CKD, chronic kidney disease; RRT, renal replacement therapy.

Between 2022 and 2027, the total healthcare cost of individuals with diagnosed CKD pre-RRT is predicted to decrease slightly from $73.2 million to $62.2 million (Figure 5a, Table S7). In 2027, CKD stage 3 is predicted to represent $50.4 million (80.9%) of the total healthcare costs, with the cost of CKD stage 3a expected to rise from $24.0 million in 2022 to $30.7 million in 2027.

The cost of RRT alone is projected to rise by 25.2%, resulting in an increase of 23.1% in the total healthcare cost of individuals with diagnosed CKD (including RRT) from $1.41 billion in 2022 to $1.73 billion in 2027 (Figure 5b, Table S7). Based on the healthcare expenditure in 2020, this will make up 2.58% of Poland’s total annual healthcare cost, with 2.48% ($1.67 billion) of this cost being attributable to RRT in 2027 (Table S7). Of the RRT healthcare costs in 2027, haemodialysis costs are predicted to make up 85.4% ($1.43 billion).

The healthcare costs of cardiovascular complications (heart failure, myocardial infarction, or stroke) for individuals diagnosed with CKD are expected to decrease slightly between 2022 and 2027, reaching $9.52 billion in 2027 (Figure S1, Table S8).

## 4. Discussion

The Inside CKD study was designed to evaluate the clinical and economic burdens of CKD between 2022 and 2027 in 31 countries. The major finding of this microsimulation projected that the prevalence of CKD in Poland will increase annually despite an overall decrease in the Polish population. Based on current practices, a high percentage of individuals with CKD will remain undiagnosed; mainly those with earlier stages of the disease (CKD stages 1–3). This increase in prevalence of CKD is reflected in the economic burden on the Polish healthcare system, where the total healthcare cost of individuals with diagnosed CKD is expected to increase by 23.1% between 2022 and 2027.

Our study projects that 12.7% of those aged ≥18 years have CKD in Poland in 2022, consistent with the 12.8% prevalence of CKD reported for Central and Eastern Europe in the Global Burden of Disease Study 2017 [9]. Further, this study highlights that should practice not improve, CKD will remain underdiagnosed in Poland. This important issue was emphasized in a recent call to action by a group of experts representing many countries in Central and Eastern Europe [29]. Reasons for CKD remaining undiagnosed include the lack of awareness of CKD among physicians and the lack of appropriate coding of CKD diagnoses in the digital documentation system. Thus, of the 38 million individuals in Poland, potentially 4 million individuals may be living with CKD, while only 0.21 million individuals were diagnosed with CKD according to the 2018 Polish National Health Fund database [6]. Our study projects that only 30.1% of individuals with CKD will be diagnosed in 2027. These data may seem alarming, but the problem of underdiagnosis of CKD seems to be similar even in the most economically developed countries. In addition, the UK was used as a proxy to calculate diagnosis rates in Poland, and as such may overestimate the percentage diagnosed. Recently published data from the REVEAL-CKD study, which analyzed country-specific electronic health databases and/or insurance claims databases from five countries (France, Germany, Italy, Japan, and the USA), showed that the prevalence of undiagnosed CKD in stage 3 was very high indeed, reaching almost 96% [30].

The rise in the prevalence of CKD is projected to lead to a 23.1% increase in healthcare costs, largely owing to a 25.2% rise in RRT costs. This is well above the 10.0% rise estimated in the Inside CKD study when including all 31 countries [25]. The costs associated with individuals with CKD are not always direct and can include the increased risk of managing complications such as cardiovascular disease. Initiating treatments for CKD at earlier stages can slow disease progression and reduce RRT costs and the incidence of such complications. For instance, renin–angiotensin–aldosterone system inhibitors have been shown to reduce mortality and morbidity in individuals with heart failure with reduced ejection fraction, and are recommended as treatment for those with cardiovascular diseases, type 2 diabetes, and CKD, while sodium–glucose cotransporter-2 inhibitors have been shown to reduce the incidence of cardiovascular events, hospitalization for heart failure, and all-cause mortality, in addition to protecting the kidneys [31,32].

The inclusion of Poland in the Inside CKD study was challenging, because forecasting the clinical and economic burdens of CKD in Central and Eastern Europe is hindered by the accurate assessment and/or recording of CKD diagnoses. Further, this part of Europe is experiencing rapid population decline, with the population of Poland expected to decrease by at least 12.0% during the next 30 years [33]. However, the population of Poland is also ageing rapidly, and this is likely to increase the incidence and prevalence of CKD [34]. This is not unexpected since the recent global analysis showed that if the 1990–2016 trend of increasing CKD prevalence and population ageing continues, the prevalence of CKD stages 3–5 may exceed 10% by 2050 in many world regions, including Central and Eastern Europe [35]. This trend may be offset by the large influx of migrants from Ukraine since 2022, consisting mainly of young women and children, whose incidence of CKD is very low. In fact, a recent report by the Renal Disaster Relief Task Force of the European Renal Association identified less than 300 Ukrainian individuals who started or continued treated in dialysis centres in Poland [36]. This indicates that immigration has had a very small (less than 1.5%) impact on the number of patients on dialysis in Poland, and therefore on the findings of this study [37].

This study provides a valuable outlook on the future burden of CKD in Poland, a country where epidemiological data are scarce. In three studies reporting on the epidemiology of CKD in Poland in the last two decades, the methodology and the age range of the sample population differed, resulting in widely varying CKD prevalence estimates [5,38,39]. For instance, the NATPOL2011 survey [5] showed a CKD prevalence of 5.8% while the PolNef study [38] showed a CKD prevalence of 11.9%. This data scarcity was also a limitation of the Inside CKD study, since the microsimulation required demographic and epidemiological data to generate the virtual population. Where data were unavailable, data from other countries considered to be the closest matches were used as a proxy. However, data obtained through the proxies were thoroughly reviewed by clinical experts in the country before being incorporated into the microsimulation model.

Overall, this study highlights the need for strategies to improve patient quality of life and reduce the future economic burden of CKD on the Polish healthcare system, including efforts that prompt earlier diagnosis of CKD and earlier initiation of interventions. Raising awareness of CKD in healthcare professionals and the public through education programmes and local or national campaigns and implementing better protocols to streamline recording of CKD and initiating treatment by integrating clinical decision support tools into electronic health record platforms are crucial strategies that could benefit individuals with CKD in Poland [40]. The main limitation of our model is that it was based on a virtual population in which only a limited number of factors could be taken into account.

## 5. Conclusions

The Inside CKD microsimulation showed that even in a country with a rapidly ageing and declining population, such as Poland, the prevalence of CKD is expected to continue to increase between 2022 and 2027. This will impose significant clinical and economic burdens on the healthcare system. The results of our analysis indicate a clear need to shift resources towards earlier diagnosis and more frequent coding of individuals with CKD, through increased awareness of CKD among physicians, potentially requiring additional training. These efforts, combined with early lifestyle and treatment interventions, including new nephroprotective therapies, have the potential to significantly reduce the clinical burden and associated healthcare costs of CKD in Poland.

## Figures and Tables

**Table 1:** Baseline characteristics (2022) of the total population at the start of the microsimulation.

Baseline Characteristic	Total Population (*n* = 37,739,779)
Sex	
Male, n (%)	18,283,212 (48.4)
Age groups, years, n (%)	
0–17	6,849,708 (18.1)
18–34	7,426,902 (19.7)
35–64	15,909,721 (42.2)
≥65	7,553,448 (20.0)
CKD stage, *n* (%)	
Stage 1	773,400 (2.0)
Stage 2	960,191 (2.5)
Stage 3a	1,724,862 (4.6)
Stage 3b	500,306 (1.3)
Stage 4	81,463 (0.2)
Stage 5	9239 (<0.1)
CKD, *n* (%)	
Total	4,049,462 (10.7)
Diagnosed	1,211,948 (3.2)
Comorbidities, *n* (%) *	
Heart failure	692,603 (17.1)
Hypertension	2,319,040 (57.3)
Type 2 diabetes	809,364 (20.0)

* Comorbidities in the CKD population. CKD, chronic kidney disease.

## Data Availability

This study did not involve the generation or collection of new primary data. A detailed report of the data inputs used in the model is available upon request.

## Supplementary Materials

The following supporting information can be downloaded at: https://www.mdpi.com/article/10.3390/jcm14010054/s1, Figure S1. Healthcare costs of cardiovascular complications in individuals diagnosed with CKD; Table S1. Baseline input data and sources; Table S2. Annual CKD diagnosis rates by CKD stage; Table S3. RRT parameters; Table S4. Proportions of individuals with CKD and diabetes or hypertension, with different albuminuria levels; Table S5. CKD-related direct healthcare costs; Table S6. Projected all-cause deaths in individuals diagnosed with CKD, by stage and year; Table S7. Healthcare costs associated with individuals diagnosed with CKD, by year; Table S8. Healthcare costs associated with cardiovascular complications and comorbidities in individuals diagnosed with CKD, by year.
