# Supplementary material for: The Clinical and Economic Burden of Chronic Kidney Disease in Poland: Inside Patient-Level Microsimulation Modelling of CKD
Source: J Clin Med. 2024 Dec 26;14(1):54. doi: 10.3390/jcm14010054 (PMC11721912; doi:10.3390/jcm14010054)
Supplement: Supplementary file 1 [file jcm-14-00054-s001.zip › jcm-3320546-supplementary.pdf]

Supplementary Materials

The Clinical and Economic Burden of Chronic Kidney Disease in Poland: Inside CKD Patient-Level Microsimulation Modelling

Anna Masajtis-Zagajewska, Renata Kurek, Katarzyna Modrzyńska, Timothy Coker and Michał Nowicki

Figure S1. Healthcare costs of cardiovascular complications in individuals diagnosed with CKD.
CKD, chronic kidney disease; RRT, renal replacement therapy.

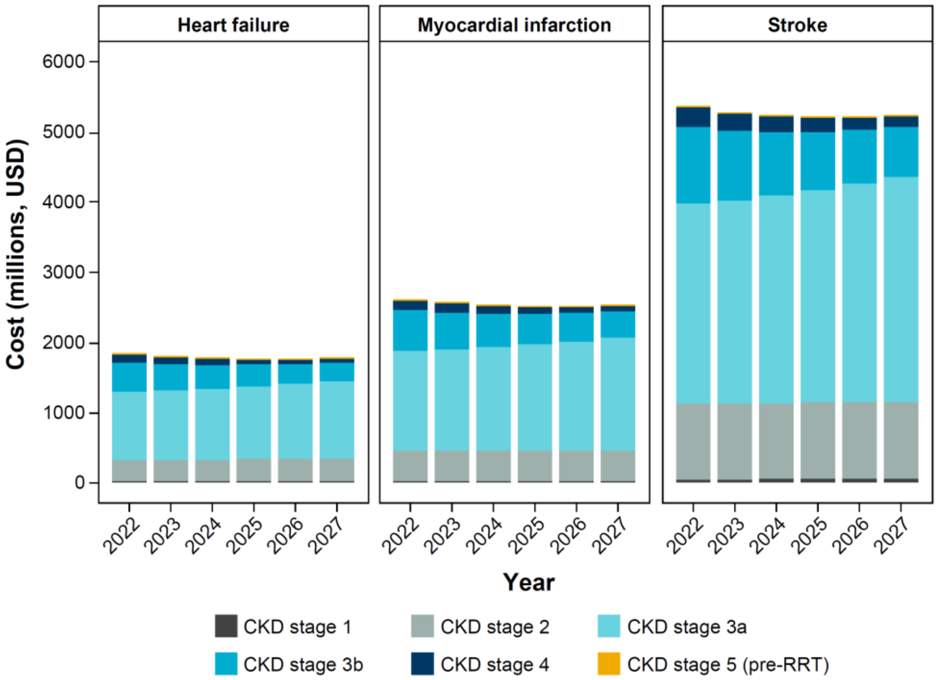

Table S1. Baseline input data and sources.

Parameter	Reference
**Population**	
Population estimates<br>Population projection<br>Total fertility rate<br>Births by mothers' age	The United Nations, World Population Prospects 2019 [1]
**CKD cohort**	
eGFR	Zdrojewski et al., 2015 [2]; Chudek et al., 2014 [3]
AER	Zdrojewski et al., 2015 [2]; Chudek et al., 2014 [3]
CKD diagnosis rates	Hirst et al. 2020 [4]; Health Survey for England, 2016 [5]
RRT parameters	Myśliwiec 2021 [6]; National Health Fund CKD, 2020 [7]; Dębska-Ślizień et al., 2022 [8]; ANZDATA 43rd Annual Report 2020 [9]
**Disease burden**	
Proportion of CKD with hypertension; diabetes	Health Survey for England, 2016 (UK proxy) [5]; Barzilay et al., 2018 (UK proxy) [10]
Heart failure incidence; prevalence; relative risk	MPZ Kardiologia 2018 [11]; National inpatient claims database analysis (data on file), Romania proxy; USRDS, 2009 [12]
MI incidence; prevalence; relative risk	National inpatient claims database analysis (data on file), Romania proxy; Dutch Heart Foundation, 2021 [13], Netherlands proxy; USRDS, 2009 [14]
Stroke incidence; prevalence; relative risk	National inpatient claims database analysis (data on file), Romania proxy; Dutch Heart Foundation, 2021 [13], Netherlands proxy; Masson et al., 2015 [14]
**Costs**	
Costs by CKD stages	AEK Forxiga CKD analysis, 2021 [15]
Costs of dialyses, transplants, heart failure events	AEK Forxiga CKD analysis, 2021 [15]; Hałdaś et al., 2015 [16]; Stafylas et al., 2017 [17], (Greece proxy); Maniadakis et al., 2013 [18] (Greece proxy)
Total healthcare expenditure in 2020	National Health Fund, 2020 [7]

AER, albumin excretion rate; CKD, chronic kidney disease; eGFR, estimated glomerular filtration rate; MI, myocardial infarction; RRT, renal replacement therapy; USRDS, U.S. Renal Data System.

Table S2. Annual CKD diagnosis rates by CKD stage.

CKD stage	Diagnosis rate (%)
Stage 1	4.3
Stage 2	27.2
Stage 3a	36.8
Stage 3b	39.6
Stage 4	73.5
Stage 5	100.0

Note, an average of values reported by Hirst et al. 2020 [4] and the Health Survey for England, 2016 [5] were used to calculate the diagnosis rates. CKD, chronic kidney disease.

Table S3. RRT parameters.

Parameter	Assumption	Reference
RRT initiation threshold (mL/min/1.73m^2)	<15	Myśliwiec 2021 [6]
Percent chance of being treated each year (%)	100	National Health Fund CKD, 2020 [7]; Dębska-Ślizień et al, 2020 [8]
Percentage of individuals with incident RRT who are on haemodialysis (%)	81.25	
Percentage of individuals with incident RRT who are on peritoneal dialysis (%)	4.59	
Percentage of individuals with prevalent RRT who are on haemodialysis (%)	68.82	
Percentage of individuals with prevalent RRT who are on peritoneal dialysis (%)	3.12	
Transplant rate per hundred individuals on dialysis per year (%)	3.31	
Annual risk of transplant failure (%)	2.6	ANZDATA, 2020 [9] (Australia proxy)

CKD, chronic kidney disease; RRT, renal replacement therapy.

Table S4. Proportions of individuals with CKD and diabetes or hypertension, with different albuminuria levels.

Parameter	Albuminuria level	Value (%)	Reference
The proportion of individuals with CKD and diabetes by albuminuria category	Normal albuminuria	19	Health Survey for England, 2016 [5] (UK proxy)
	Microalbuminuria	20	
	Macroalbuminuria	27	
The proportion of individuals with CKD with hypertension by albuminuria category	Normal albuminuria	65	Health Survey for England, 2016 [5]; Barzilay et al., 2018 [10] (UK proxy)
	Microalbuminuria	43	
	Macroalbuminuria	80	

CKD, chronic kidney disease.

Table S5. Chronic kidney disease-related direct healthcare costs.

Condition	Cost in 2022* ($)	Reference
**Median cost per individual by CKD stage and RRT**		
CKD stage 1	Assumed 0	–
CKD stage 2	Assumed 0	–
CKD stage 3a	38	AEK Forxiga CKD analysis, 2021 [15]
CKD stage 3b	152	
CKD stage 4	305	
CKD stage 5 (pre-RRT)	343	
Haemodialysis	39 092	
Peritoneal dialysis	50 121	
Kidney transplant first year	37 449	
Kidney transplant subsequent years	9633	Hałdaś et al., 2015 [16]
**Mean cost per individual by complications**		
Heart failure	8633	Stafylas et al., 2017 [17] (Greece proxy)
Myocardial infarction	10 248	Maniadakis et al., 2013 [18] (Greece proxy)
Stroke	8579	Hałdaś et al., 2015 [16]

*Inflated from the year reported to 2022 according to GDP deflator data from the IMF-WEO report accessed Oct 2022 [19].
CKD, chronic kidney disease; GDP, gross domestic product; IMF, International Monetary Fund; RRT, renal replacement therapy; WEO, world economic outlook.

Table S6. Projected all-cause deaths in individuals diagnosed with CKD, by stage and year.

CKD stage	All-cause death (n)					
	Year					
	2022	2023	2024	2025	2026	2027
Stage 1	547	584	595	647	783	793
Stage 2	16 989	16 410	15 741	15 241	15 131	15 797
Stage 3a	40 964	39 716	39 061	39 035	39 652	40 556
Stage 3b	27 338	24 490	22 349	20 380	19 600	18 560
Stage 4	19 933	16 122	13 984	12 621	10 516	8560
Stage 5	3265	2482	2345	2236	2530	3209
Total	109 036	99 804	94 074	90 160	88 212	87 474

CKD, chronic kidney disease.

Table S7. Healthcare costs associated with individuals diagnosed with CKD, by year.

CKD stage	Cost ($, millions)						
	Year						Difference in 2022–2027 (%)
	2022	2023	2024	2025	2026	2027	
**Stage 3–5 (pre-RRT)**	73.2	69.1	67.0	65.5	63.6	62.2	-15.0
Stage 3a	24.0	25.3	26.5	27.9	29.3	30.7	28.0
Stage 3b	30.1	27.5	25.0	22.9	21.3	19.6	-34.8
Stage 4	18.3	15.4	14.4	13.5	11.7	10.1	-44.7
Stage 5 (pre-RRT)	0.8	0.8	1.0	1.1	1.4	1.8	121.1
**RRT**	1335.9	1348.5	1378.5	1406.8	1535.2	1672.3	25.2

Haemodialysis	1143.8	1154.4	1181.1	1205.3	1311.1	1428.0	24.9
Peritoneal dialysis	64.0	65.4	67.0	69.2	77.1	86.2	34.8
Transplant	128.2	128.7	130.4	132.2	147.0	158.1	23.3
**Total**	1409.1	1417.6	1445.5	1472.3	1598.8	1734.6	23.1

CKD, chronic kidney disease; RRT, renal replacement therapy.

Table S8. Healthcare costs associated with cardiovascular complications and comorbidities in individuals diagnosed with CKD, by year.

**Cost ($, millions)**						
**Cardiovascular complication or comorbidity**	**Year**					
	**2022**	**2023**	**2024**	**2025**	**2026**	**2027**
**Cardiovascular complications**	9771.1	9595.4	9504.5	9480.3	9486.9	9518.4
Heart failure	1811.4	1774.5	1756.3	1752.4	1752.5	1760.6
Myocardial infarction	2604.0	2553.6	2523.9	2513.6	2513.4	2525.2
Stroke	5355.7	5267.4	5224.3	5214.3	5221.0	5232.6
**Comorbidities**	50.6	47.7	46.2	45.2	43.9	42.9
Hypertension only	37.2	35.2	34.3	33.6	32.7	32.1
Type 2 diabetes only	5.1	4.7	4.5	4.3	4.1	4.0
Hypertension and Type 2 diabetes	8.3	7.8	7.5	7.2	7.0	6.8

CKD, chronic kidney disease.

References

1. World Population Prospects, Poland. Available online: https://population.un.org/wpp/Download/Standard/Population/ (accessed on July 2022).
2. Zdrojewski, Ł.; Zdrojewski, T.; Rutkowski, M.; Bandosz, P.; Król, E.; Wyrzykowski, B.; Rutkowski, B. Prevalence of chronic kidney disease in a representative sample of the Polish population: results of the NATPOL 2011 survey. *Nephrol Dial Transplant* **2016**, *31*, 433-439.
3. Chudek, J.; Wieczorowska-Tobis, K.; Zejda, J.; Broczek, K.; Skalska, A.; Zdrojewski, T.; Wiecek, A. The prevalence of chronic kidney disease and its relation to socioeconomic conditions in an elderly Polish population: results from the national population-based study PolSenior. *Nephrol Dial Transplant* **2014**, *29*, 1073-1082.
4. Hirst, J.A.; Hill, N.; O'Callaghan, C.A.; Lasserson, D.; McManus, R.J.; Ogburn, E.; Mena, J.M.O.; Shine, B.; Taylor, C.J.; Vazquez-Montes, M.D.; et al. Prevalence of chronic kidney disease in the community using data from OxRen: a UK population-based cohort study. *Br J Gen Pract* **2020**, *70*, e285-e293.
5. NatCen Social Research and UCL. *Health Survey for England, 2016*; 2017; Available online: https://digital.nhs.uk/data-and-information/publications/statistical/health-survey-for-england/health-survey-for-england-2016 (accessed on July 2022).
6. Chronic renal failure. Available online: https://www.mp.pl/pacjent/nefrologia/choroby/chorobyudoroslych/51919,przewlekla-niewydolnosc-nerek. (accessed on July 2022).
7. Chronic kidney disease. Available online: https://ezdrowie.gov.pl/5668 (accessed on July 2022).
8. Dębska-Ślizień, A.; Rutkowski, B.; Jagodziński, P.; Rutkowski, P.; Przygoda, J.; Lewandowska, D.; Czerwiński, J.; Kamiński, A.; Gellert, R. Aktualny stan dializoterapii w polsce – 2020. *Nefrol Dial Pol* **2021**, *25*, 7-20.
9. ANZDATA. *Anzdata 43rd Annual Report 2020 (Data to 2019)*; Available online: https://www.anzdata.org.au/report/anzdata-43rd-annual-report-2020-data-to-2019/ (accessed on June 2022).
10. Barzilay, J.I.; Davis, B.R.; Pressel, S.L.; Ghosh, A.; Rahman, M.; Einhorn, P.T.; Cushman, W.C.; Whelton, P.K.; Wright, J.T., Jr. The Effects of eGFR Change on CVD, Renal, and Mortality Outcomes in a Hypertensive Cohort Treated With 3 Different Antihypertensive Medications. *Am J Hypertens* **2018**, *31*, 609-614.
11. MPZ Kardiologia. *Health needs map in the field of cardiology for the Masovian Voivodeship*; 2018; Available online: https://mpz.mz.gov.pl/wp-content/uploads/2019/06/mpz_kardiologia_mazowieckie.pdf (accessed on July 2022).
12. United States Renal Data System. *USRDS 2009 Annual Data Report: Atlas of Chronic Kidney Disease and End-Stage Renal Disease in the United States*; U.S. Renal Data System: 2009; Available online: http://www.usrds.org/atlas09.aspx (accessed on July 2020).
13. Dutch Heart Foundation. *Cardiovascular disease in the Netherlands, 2021*; Available online: https://www.hartstichting.nl/ (accessed on August 2022).
14. Masson, P.; Webster, A.C.; Hong, M.; Turner, R.; Lindley, R.I.; Craig, J.C. Chronic kidney disease and the risk of stroke: a systematic review and meta-analysis. *Nephrol Dial Transplant* **2015**, *30*, 1162-1169.
15. AEK Forxiga. *Dapagliflozin (Forxiga) in the treatment of chronic kidney disease: Economic Analysis*; 2021; Available online: https://bipold.aotm.gov.pl/assets/files/zlecenia_mz/2021/181/AW/181_OT.4230.22.2021_Forxiga_AE.pdf (accessed on July 2022).
16. Hałdaś, M.; Chudzicka, A.; Ziobro, M.; Rutkowski, J.; Glasek, M.; Lis, J. Reaching Therapeutic Goals Impacts on Estimated Cost of Illness for Patients with Type 2 Diabetes in Poland. *Journal of Health Policy and Outcomes Research* **2015**, *1*, 44-55.

17. Stafylas, P.; Farmakis, D.; Kourlaba, G.; Giamouzis, G.; Tsarouhas, K.; Maniadakis, N.; Parissis, J. The heart failure pandemic: The clinical and economic burden in Greece. *Int J Cardiol* **2017**, *227*, 923-929.
18. Maniadakis, N.; Kourlaba, G.; Cokkinos, D.V.; Angeli, A.; Kyriopoulos, J. The economic burden of atherothrombosis in Greece: results from the THESIS study. *Eur J Health Econ* **2013**, *14*, 655-665.
19. International Monetary Fund. *World Economic Outlook Report*; 2022; Available online: https://www.imf.org/en/Publications/WEO/Issues/2022/10/11/world-economic-outlook-october-2022 (accessed on October 2022).
